# Complete Chloroplast Genome Sequence of Chinese Lacquer Tree (*Toxicodendron vernicifluum*, Anacardiaceae) and Its Phylogenetic Significance

**DOI:** 10.1155/2020/9014873

**Published:** 2020-01-30

**Authors:** Lu Wang, Na He, Yao Li, Yanming Fang, Feilong Zhang

**Affiliations:** ^1^Co-Innovation Center for Sustainable Forestry in Southern China, College of Biology and the Environment, Key Laboratory of State Forestry and Grassland Administration on Subtropical Forest Biodiversity Conservation, Nanjing Forestry University, Nanjing 210037, China; ^2^Xi'an Raw Lacquer and Research Institute, Xi'an 710061, China

## Abstract

Chinese lacquer tree (*Toxicodendron vernicifluum*) is an important commercial arbor species widely cultivated in East Asia for producing highly durable lacquer. Here, we sequenced and analyzed the complete chloroplast (cp) genome of *T. vernicifluum* and reconstructed the phylogeny of Sapindales based on 52 cp genomes of six families. The plastome of *T. vernicifluum* is 159,571 bp in length, including a pair of inverted repeats (IRs) of 26,511 bp, separated by a large single-copy (LSC) region of 87,475 bp and a small single-copy (SSC) region of 19,074 bp. A total of 126 genes were identified, of which 81 are protein-coding genes, 37 are transfer RNA genes, and eight are ribosomal RNA genes. Forty-nine mononucleotide microsatellites, one dinucleotide microsatellite, two complex microsatellites, and 49 long repeats were determined. Structural differences such as inversion variation in LSC and gene loss in IR were detected across cp genomes of the six genera in Anacardiaceae. Phylogenetic analyses revealed that the genus *Toxicodendron* is closely related to *Pistacia* and *Rhus*. The phylogenetic relationships of the six families in Sapindales were well resolved. Overall, this study providing complete cp genome resources will be beneficial for determining potential molecular markers and evolutionary patterns of *T. vernicifluum* and its closely related species.

## 1. Introduction

*Toxicodendron vernicifluum* (Stokes) F. A. Barkley, commonly known as the Chinese lacquer tree, is a deciduous tree species with a toxic sap in the sumac family, Anacardiaceae [1]. The generic name of the species is derived from the Greek words *toxikos*, meaning “poison,” and *dendron*, meaning “tree,” while the specific name *vernicifluum* means “lacquer” in Latin [2]. *T. vernicifluum* is native to China and the Indian subcontinent and has been cultivated in other oriental countries, such as South Korea and Japan, for probably thousands of years [3–5]. Through tapping the trunk, the species can provide us with the raw lacquer, an excellent adhesive and painting material with multiple characteristics, such as anticorrosion, antirust, nonoxidation, acid resistance, alcohol resistance, and high-temperature resistance [3, 6]. The lacquer is traditionally used to make various types of lacquerwares in China, Japan, South Korea, and several countries in Southeast and South Asia [1]. Furthermore, *T. vernicifluum* is sometimes used in Chinese medicine for the treatment of internal parasites and for stopping bleeding. Previous studies also reported that the urushiols of the species probably have an anticancer activity to human cancer cells, and the flavonoids extracted from its leaves possess therapeutic potentials as a multipotent agent against neurodegenerative diseases [7, 8].

Many recent studies of the Chinese lacquer tree have focused on secondary metabolites [9–11], anatomic features [12–14], growth traits [15–17], and classification and evaluation of cultivars of the species [18, 19]. Since 1978, more than 90 local cultivars, including some rare ones, have been recognized [20]. Those cultivars have different properties, such as high yields, good quality of lacquer, and good timber [21]. However, controversy still surrounded the delimitation of the cultivars based on morphological and anatomic traits [22]. Although some researchers have used different types of molecular markers, such as nuclear microsatellites (nSSRs) and amplified fragment length polymorphism (AFLP), to describe the genetic variation of both natural populations and cultivars of *T. vernicifluum* [23, 24], the information on the genetic diversity and structure of the species was still limited by small sample size, narrow sampling range, and a few number of molecular markers. Thus, more studies on both nuclear and plastid genomes are expected to provide more useful markers to reveal the genetic variation pattern of *T. vernicifluum* and its cultivars in the future.

The genus *Toxicodendron* Mill. is well known for possessing urushiols, which can cause severe allergic contact dermatitis [25]. It consists of approximately 24 species with a disjunct distribution in temperate North America and eastern Asia [26]. Fifteen of them are native to China, mainly distributed in the regions south of the Yangtze River [27]. Previous studies have shown that *Toxicodendron* is a monophyletic group distinct from other genera of the *Rhus* complex [28]. Two temperate disjunct lineages have been recovered, one from section *Toxicodendron* and the other between the eastern North American *T. vernix* and the eastern Asian *T. vernicifluum*. The biogeographic history of the genus suggested that the Bering land bridge may have acted as the migration route that resulted in the current pattern of temperate disjunctions. Nonetheless, intrageneric relationships of *Toxicodendron* are still poorly understood [28]. Furthermore, Anacardiaceae is among the families of Sapindales, which is known for citrus, maples, lychees, mangos, and cashews [29]. Previous studies have shown that Sapindales is a monophyletic group [30, 31]. However, phylogenetic relationships among several families of the order, such as Simaroubaceae, Rutaceae, and Meliaceae, are still not fully resolved [30–32].

The chloroplast (cp) genome is nonrecombinant and uniparental and is more conserved than mitochondrial and nuclear genomes in terms of gene content, organization, and structure [33]. Also, the nucleotide substitution rate of chloroplast genes is higher than those of mitochondria genome but lower than those of nuclear genome [34]. The cp genome plays an important role in reconstruction of the green plant phylogeny and understanding the origins of economically important cultivated species and changes that have taken place during domestication [35, 36]. With the development of bioinformatics and high-throughput sequencing technology, various studies on the evolution of cp genomes have emerged in recent years [37].

Recently, the cp genome of *T. vernicifluum* cv. *Dahongpao*, a natural triploid cultivar, has been reported [38]. However, the differences of plastomes between diploid and triploid are rarely known. In this study, we sequenced and analyzed the complete cp genome of diploid *T. vernicifluum* and reconstructed the phylogeny of Sapindales based on 52 cp genomes of six families. The following questions were addressed: (1) What are the features of the cp genome of diploid *T. vernicifluum*? (2) How many potential microsatellite markers can the cp genome provide for us? (3) What kinds of structural variation events have occurred across the cp genomes in Anacardiaceae? and (4) Can the cp genome information provide supporting data for phylogenetic reconstruction of both Anacardiaceae and Sapindales?

## 2. Materials and Methods

### 2.1. Sampling, DNA Extraction, and Illumina Sequencing

Healthy and fresh leaves of diploid *T. vernicifluum* were collected from an adult tree growing in the Lacquer Paint Research Institute, Shaanxi Province, China, during September 2018. The voucher specimen of the individual (voucher accession number LW20180905001) was stored at the Herbarium of Lacquer Paint Research Institute. Total genomic DNA was extracted using a modified CTAB method [39] and was fragmented by mechanical interruption (ultrasonic). After quality testing, the PCR-amplified library was sequenced with the Illumina Hiseq X Ten platform (Illumina Inc., San Diego, CA, United States) according to the manufacturer's manual.

### 2.2. Chloroplast Genome Assembling and Annotation

The NOVOplasty ver. 2.7.2 software [40] was used for the *de novo* assembly of the chloroplast genome of *T. vernicifluum* based on the cp-like reads extracted from a total of 25,291,737 high-quality sequences (Phred score ≥30) generated by Illumina sequencing. The CpGAVAS pipeline [41] was applied to annotate the protein-coding, rRNA, and tRNA genes. The tRNAscan-SE ver. 1.21 software [42] was used to verify the tRNA genes with default settings. The circular gene map was drawn by the OrganellarGenomeDRAW tool (OGDRAW) ver. 1.3.1 [43]. The relative synonymous codon usage (RSCU) value was estimated for each codon based on the coding sequences of 81 protein-coding genes using Phylosuite ver. 1.1.152 [44]. Simple sequence repeats (SSRs) across the cp genome were determined by MISA (http://pgrc.ipk-gatersleben.de/misa/misa.html) [45], with the minimum number of repeats set to 10 for mononucleotide, to 6 for dinucleotide, and to 5 for tri-, tetra-, penta-, and hexanucleotide SSRs. We also used REPuter (https://bibiserv.cebitec.uni-bielefeld.de/reputer/) [46] to identify forward, reverse, complement, and palindromic repeats, with the minimum repeat size set to 8 and the hamming distance set to 1.

### 2.3. Genome Comparison

We used the publicly available chloroplast genome sequences of species from five genera of Anacardiaceae (last accessed 1 April 2019), i.e., *Rhus chinensis* Mill. (GenBank accession number KX447140), *Pistacia weinmannifolia* J. Poiss. ex Franch. (MF630953), *Anacardium occidentale* L. (KY635877), *Mangifera indica* L. (KY635882), and *Spondias mombin* L. (KY828469) to perform the comparative cp genomic analysis with *T. vernicifluum*. The sequence identity of those genomes was plotted using the mVISTA program [47] with LAGAN mode. Multiple genome alignments were conducted through the progressive Mauve algorithm [48] as implemented in the Geneious software (Biomatters, Auckland, New Zealand) to detect the presence of large-scale evolutionary events such as rearrangement and inversion. The borders of large single-copy (LSC), small single-copy (SSC), and inverted repeat (IR) regions were visually displayed and compared among the six species using Irscope [49]. To detect the hotspots of intergeneric divergence, sequences of 100 common genes and 37 intergenic spacers were extracted for each species using Phylosuite ver. 1.1.152 [44] and aligned by MAFFT ver. 7.313 [50]. Following this step, the nucleotide diversity (*P*_i_) value was calculated for each of the 137 loci using DnaSP ver. 6.12.03 [51].

### 2.4. Phylogenetic Analysis

We reconstructed the phylogeny of Sapindales based on 52 cp genomes representing 38 genera of six families ([Supplementary-material supplementary-material-1]), including two genera of Burseraceae, two genera of Simaroubaceae, 10 genera of Meliaceae, six genera of Anacardiaceae, 10 genera of Sapindaceae, and nine genera of Rutaceae. Two species of Brassicales (*Carica papaya* L.) and Huerteales (*Tapiscia sinensis* Oliv.) ([Supplementary-material supplementary-material-1]) were selected as outgroups following the interrelationships of orders recognized by APG IV [52]. These genomes cannot be aligned directly due to the occurrence of numerous differences regarding gene content and structure. Therefore, we used the HomBlocks pipeline [53] that automatically recognizes locally collinear blocks (LCBs) and excavates phylogeny informative regions to construct the multigene involved alignment. In brief, the progressive Mauve algorithm [48, 54] was applied at first to identify coexisting blocks. Those LCBs were extracted and then trimmed by Gblocks [55]. The circoletto webserver (http://tools.bat.infspire.org/circoletto/) was used for the visualization of genes that were integrated into the final alignment. The best-fit partitioning schemes and DNA substitution models were determined by PartitionFinder [56] using a greedy search strategy. Finally, the GTR + G model was chosen for the subset including LCBs 2-3, and the TVM + I + G model was chosen for the subset including LCBs 4–6, the subset including LCBs 8-9, and the subset including only LCB5 ([Supplementary-material supplementary-material-1]). The phylogenetic relationships among those 54 species were reconstructed using Bayesian-inference (BI) analyses as implemented in MrBayes ver. 3.2.6 [57]. A Markov chain Monte Carlo (MCMC) was run for 2,000,000 generations with two parallel searches using four chains, each starting with a random tree. Trees were sampled every 100 generations and the first 25% was discarded as burn-in. The maximum likelihood (ML) analyses were also performed with RaxmlGUI ver. 1.5b1 [58, 59]. The GTRGAMMA model was selected for all the subsets, and branch support values were estimated for each node based on 1,000 samples for rapid bootstrap.

## 3. Results

### 3.1. Features of *T. vernicifluum* Chloroplast Genome

**Figure 1:**
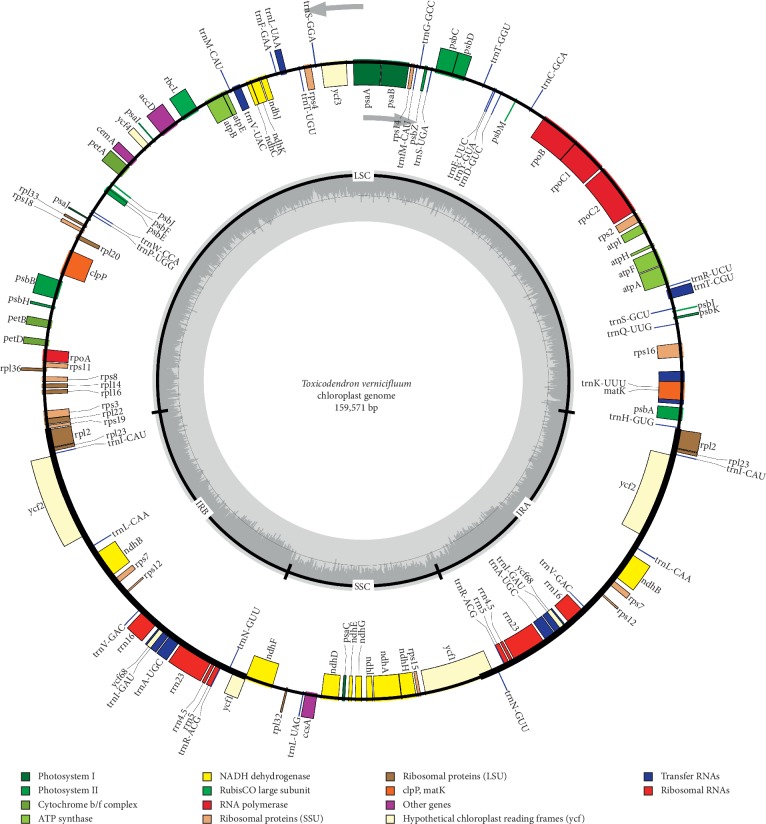
Gene map of *Toxicodendron vernicifluum* chloroplast genome. Genes shown inside the circle are transcribed clockwise and those outside are transcribed counterclockwise. Genes of different functions are color-coded. The darker gray in the inner circle shows the GC content, while the lighter gray shows the AT content.

A total of 25,291,737 paired-end reads were produced by the Illumina Hiseq X Ten sequencing platform. After *de novo* assembly, the complete cp genome sequence of *T. vernicifluum* was obtained and submitted to the NCBI database under the GenBank accession number MK419151. The cp genome of *T. vernicifluum* is a circular molecule of 159,571 bp, consisting of a large single-copy (LSC) region of 87,475 bp, a small single-copy (SSC) region of 19,074 bp, and a pair of inverted repeats (IRa and IRb) of 26,511 bp (Table 1 and Figure 1). The base composition of the complete cp genome sequence was analyzed and found to be 31.30% T, 30.74% A, 19.06% C, and 18.90% G. The overall GC content was 37.96%, which is very close to those of other Anacardiaceae species, e.g., *M. indica* (37.89%), *R. chinensis* (37.79%), and *A. occidentale* (38.12%) (Table 1) and also to those of other species in Sapindales, e.g., *Toona ciliata* M. Roem. (37.90%) [60], *Commiphora gileadensis* (L.). C. Chr. (37.90%) [61], and *Xanthoceras sorbifolium* Bunge (37.70%) [62]. Furthermore, the GC contents are unevenly distributed across regions of the cp genome, which were found to be 36.08%, 32.63%, and 42.96% for the LSC, SSC, and IR regions, respectively (Table 2).

The complete cp genome of *T. vernicifluum* encodes 126 predicted functional genes, including 81 protein-coding genes, 37 transfer RNA (tRNA) genes, and eight ribosomal RNA (rRNA) genes ([Supplementary-material supplementary-material-1]). Among those, 107 are unique, and 19 are duplicated in the IR regions. For the 107 unique genes, 30 are tRNA genes, four are rRNA genes, and 73 are protein-coding genes. For the 19 duplicated genes, eight are protein-coding genes, seven are tRNA genes, and four are rRNA genes. Within the cp genome of *T. vernicifluum*, 12 genes, including six tRNA genes and six protein-coding genes, contain only one intron. Two genes (*ycf*3 and *clp*P) contain two introns ([Supplementary-material supplementary-material-1]).

### 3.2. Codon Usage Bias

The relative synonymous codon usage (RSCU) value was estimated for each codon based on the coding sequences (CDS) of 81 protein-coding genes, which presented a total length of 80,280 bp and accounted for 50.31% of the complete cp genome of *T. vernicifluum*. A total of 26,760 codons were found in those coding regions. The most prevalent amino acid is leucine (2,794 codons, approximately 10.44%), followed by isoleucine (2,249 codons, approximately 8.40%) and serine (2,107 codons, approximately 7.87%), while the rarest one is cysteine (318 codons, approximately 1.19%) ([Supplementary-material supplementary-material-1]). In addition, almost all of the A/U-ending codons showed RSCU values greater than 1, whereas the C/G-ending codons showed RSCU values less than 1. For example, synonymous codons GUU, GUC, GUA, and GUG encode valine, and the corresponding RSCU values for these four codons are 1.43, 0.51, 1.49, and 0.57, respectively, as expected from the low GC content of CDS.

### 3.3. Repeat Sequences

Simple sequence repeats (SSRs) are DNA stretches consisting of short, tandemly repeated motifs with a length of 1–6 bp, which have been widely used as molecular markers in population genetics and evolutionary biology [63–65]. In this study, a total of 52 chloroplast SSRs (cpSSRs) were identified ranging in length from 10 to 78 bp. Among those, 44 are distributed in intergenic spacers (IGSs), six are located at coding regions of three genes (*rpo*C2, *atp*B, and *ycf*1), and only two are within the introns of *ycf*3 and *clp*P ([Supplementary-material supplementary-material-1]). On the other hand, most SSRs are distributed in LSC and SSC, whereas only 11 are located at IR regions ([Supplementary-material supplementary-material-1]).

Mononucleotide SSRs were found to be the richest, with 49 of 52 belonging to this type. One of the other three is dinucleotide microsatellite with (AT)_*n*_ repeats, and two are complex microsatellites that include a small insertion and/or contain two different types of repeats ([Supplementary-material supplementary-material-1]). No tri-, tetra-, penta-, and hexanucleotide SSRs were detected. Among the mononucleotide SSRs, polyadenine (poly A) and polythymine (poly T) occupy the highest portion (91.84%) and mononucleotide C and G are rare (8.16%). The higher level of A or T within those cpSSRs is consistent with the overall A/T content (62.04%) of the cp genome of *T. vernicifluum*. These SSR loci could be used for investigations into genetic diversity and genetic structure of natural populations and cultivars of *T. vernicifluum*.

Long repeats may promote the cp genome rearrangement and increase the population genetic diversity [66]. Using REPuter (https://bibiserv.cebitec.uni-bielefeld.de/reputer), 49 long repeats were identified across the *T. vernicifluum* plastome, ranging from 22 to 66 bp in length ([Supplementary-material supplementary-material-1]). Among those, 23 are forward repeats, 22 are palindromic repeats, and four are reverse repeats. Multiple nested sequence repeats were detected in *ycf*2.

### 3.4. Genome Comparison of Anacardiaceae Species

**Figure 2:**
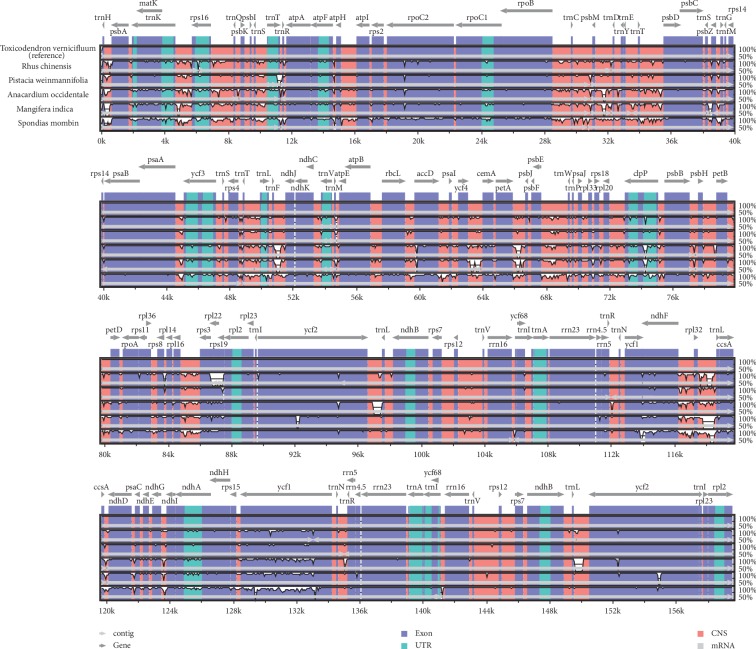
Visualization of alignment of the six Anacardiaceae species using mVISTA, with *T. vernicifluum* as the reference. The horizontal axis indicates the coordinates within the chloroplast genome. The vertical scale represents the percentage of identity, ranging from 50% to 100%. The gray arrows above the alignment indicate the genes' orientations.

To investigate the intergeneric divergence of cp genome sequences, the percentage of identity was plotted for six species from different genera in Anacardiaceae using the mVISTA program with *T*. *vernicifluum* as the reference. High similarity was detected among those six genera, and the IR regions were found to be more conserved than the LSC/SSC regions (Figure 2). Furthermore, the variation of noncoding regions is significantly higher than that of coding regions (Figure 2).

**Figure 3:**
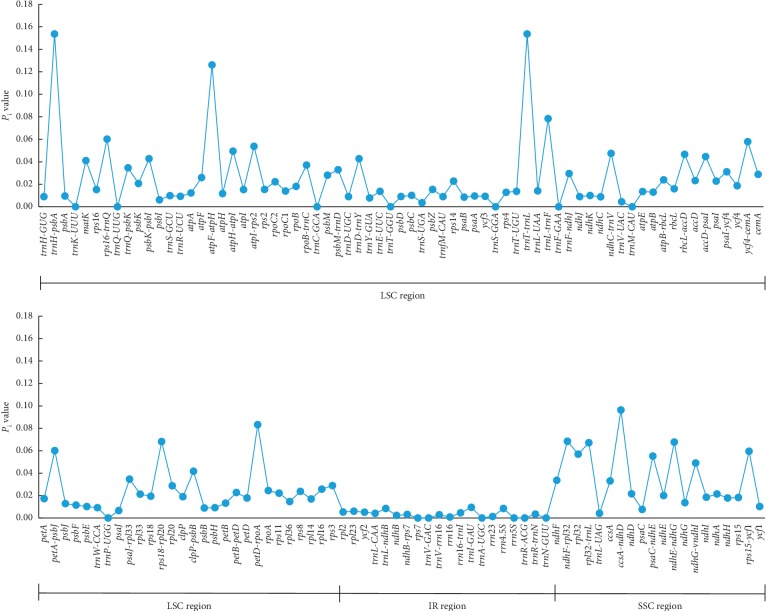
Nucleotide diversity (*P*_i_) values among the six Anacardiaceae species.

The nucleotide diversity (*P*_i_) values were calculated for 137 loci (100 genes and 37 intergenic spacers) to determine the hotspots of divergence. Those values ranged from 0 to 0.154, with a higher level of genetic variation detected within the LSC (average *P*_i_=0.025) and SSC regions (average *P*_i_=0.037) than within IR regions (average *P*_i_=0.003) (Figure 3). In addition, intergenic spacers (average *P*_i_=0.053) were found to be more variable than genes (average *P*_i_=0.013) (Figure 3). Six of these loci, including *trn*H-*psb*A (0.154), *trn*T-*trn*L (0.154), *atp*F-*atp*H (0.126), *ccs*A-*ndh*D (0.096), *pet*D-*rpo*A (0.083), and *trn*L-*trn*F (0.078), showed high levels of nucleotide diversity (*P*_i_ > 0.070) across the six genera of Anacardiaceae (Figure 3).

**Figure 4:**
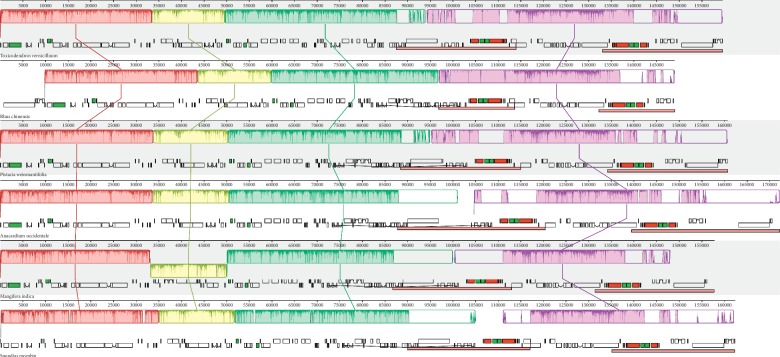
Gene map and MAUVE alignment of six Anacardiaceae chloroplast genomes. Within each of the alignment, local collinear blocks are represented by blocks of the same color connected by lines.

To characterize the structure and synteny, the cp genome sequences of the six Anacardiaceae species were aligned by Mauve, and *T. vernicifluum* was used as a reference to compare the gene orders among these cp genomes (Figure 4). Synteny analysis indicated that no rearrangement events were identified. However, differences were still found in terms of inversion variation in LSC and gene loss in IRs, leading to the occurrence of four collinearity modules across the six cp genome sequences. First, a large inversed segment was detected for *M. indica*, which resulted in the separation of the first block from the third block. This inversion was between *trn*E-UUC and *trn*L-UAA, with a length of 16,910 bp (ranging from 33,130 to 50,039 bp). Fourteen genes were included in this fragment, and two tRNA genes (*trn*T-GGU and *trn*T-UGU) were observed at the two ends. Second, due to the loss of a large fragment (∼9,800 bp) in the IRb region of *R. chinensis*, the remaining part of the cp genome was divided into two blocks (the third and the fourth blocks). Instead, if the plastome of *R. chinensis* was removed, only three collinearity modules were identified across the cp genome sequences of the rest five species ([Supplementary-material supplementary-material-1]). This event has reduced the IR length of *R. chinensis* severely (Table 1) and shifted four genes (*ycf*2, *trn*I-CAU, *rpl*23, and *rpl*2) from IR to LSC regions.

### 3.5. IR Expansion and Contraction

**Figure 5:**
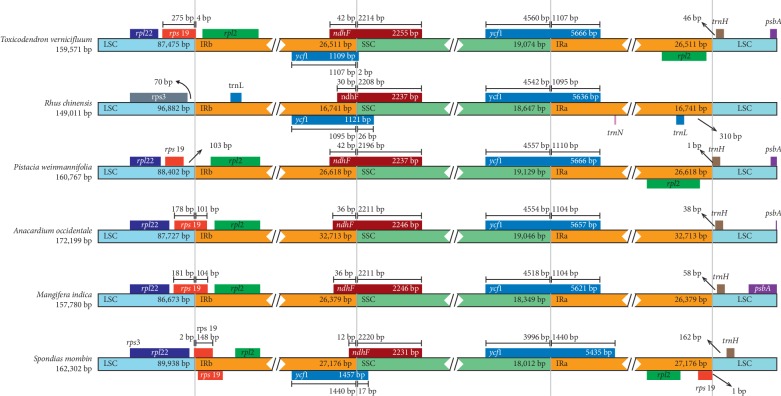
Comparison of IR-SC border positions across chloroplast genomes of six Anacardiaceae species. Genes are denoted by colored boxes. The gaps between the genes and the boundaries are indicated by the base lengths (bp).

For the six Anacardiaceae species, the length of IR was found to be significantly correlated with the total length of the complete cp genome (*R* = 0.962, *P*=0.002) ([Supplementary-material supplementary-material-1]). Among those, *R. chinensis* presented the shortest length of cp genome, which was mainly attributed to the loss of a long fragment (∼9,800 bp in length) at the IRb/LSC boundary. By contrast, *A. occidentale* showed the longest length of cp genome, which was associated with the size expansion of *ycf*2-*trn*L spacer (∼6,200 bp longer than the other five species) within the IR regions (Table 1). Furthermore, minor shifts of IR/SC boundaries were detected for those species (Figure 5). For example, The *rps*19 gene is across the IRb/LSC border with 2–275 bp extending into the LSC region, while it was found to be 103 bp away from the border of *P. weinmannifolia*. Similarly, the *trn*H gene is located in the LSC region, 38–162 bp away from the IRa/LSC border, while it extends only 1 bp into the IRa region of *P. weinmannifolia*. For all the six species, the IRa/SSC junction is located in the *ycf*1 region, and the IRb/SSC junction is located in the *ndh*F region. However, the length included in the IRs varies significantly across species, namely, 1,095–1,440 bp for *ycf*1 and 12–42 bp for *ndh*F. Previous studies have shown that pseudogene *ycf*1 overlaps with normal gene *ndh*F in the IRa/SSC border for many higher plants [67, 68]. This was observed for *T. vernicifluum*, *R. chinensis*, and *S. mombin*, but not for *P. weinmannifolia*, *A. occidentale*, and *M. indica*.

### 3.6. Phylogenetic Inference

**Figure 6:**
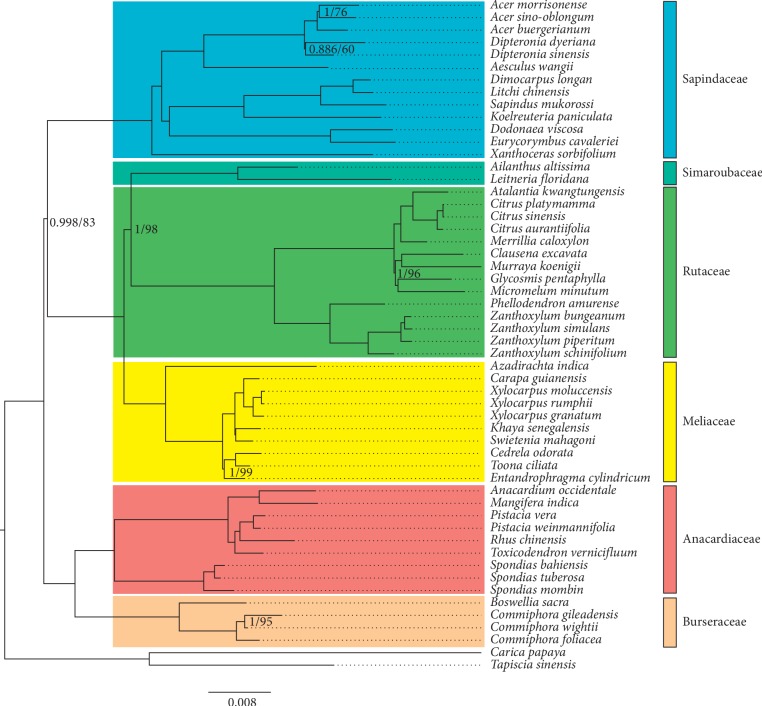
Bayesian-inference (BI) and maximum likelihood (ML) analyses based on the chloroplast genome sequences of 52 species from six families in Sapindales and two outgroups of Brassicales and Huerteales. Above each node, the first number indicates the Bayesian posterior probability (PP), and the second number indicates the ML bootstrap value (BS). Nodes with posterior probability of 1 and bootstrap value of 100 are not labeled.

Using HomBlocks, nine locally collinear blocks (LCBs) were identified across the cp genome sequences of 52 species in Sapindales, and two outgroups from Brassicales and Huerteales. After trimming, the final alignment produced a matrix of 65,219 bp ([Supplementary-material supplementary-material-1]), *including 20,974 variable sites and 13,581 parsimony-informative sites*. Most protein-coding genes have been integrated into the final alignment ([Supplementary-material supplementary-material-1]). The ML and Bayesian analyses yielded identical tree topologies, with all the nodes at or above the generic level allocated posterior probabilities (PP) ≥0.99 and bootstrap support (BS) values ≥80% (except for the genus *Dipteronia*, Figure 6). Our results showed that Anacardiaceae and Burseraceae form a monophyletic clade (1.0 PP/100 BS), and the remaining four families form another clade (0.998 PP/83 BS). Simaroubaceae and Rutaceae were resolved as sister to Meliaceae with strong support (1.0 PP/100 BS). Within the Anacardiaceae, the genus *Spondias* was found to be sister to the rest five genera (1.0 PP/100 BS), and *T. vernicifluum* was sister to the clade of *R. chinensis* and two *Pistacia* species (1.0 PP/100 BS).

### 3.7. Differences of Plastomes between Diploid and Triploid *T. vernicifluum*

The size of the cp genome (159,571 bp) was completely consistent with the result of *T. vernicifluum* cv. *Dahongpao*, a natural triploid cultivar of the species [39]. Only two single nucleotide variants were detected between the two genotypes: one located at the *rpo*B-*trn*C(GCA) spacer and another within the *ycf*1 gene region ([Supplementary-material supplementary-material-1]). No indels were observed between them.

## 4. Discussion

In this study, we sequenced the complete cp genome of the Chinese lacquer tree by using Illumina high-throughput sequencing technology. No size variation and only two single nucleotide variants were detected between diploid and triploid of *T. vernicifluum*, suggesting that the species exhibited extremely conserved genome size and structure at the intraspecific level. Compared with closely related species in Anacardiaceae, the length of the cp genome of *T. vernicifluum* is shorter than those of *A. occidentale* (172,199 bp), *S. mombin* (162,302 bp), and *P. weinmannifolia* (160,767 bp), but longer than those of *M. indica* (157,780 bp) and *R. chinensis* (149,011 bp) (Table 1). We found that the total length of the complete cp genome was significantly correlated with the length of IR (*R* = 0.962, *P*=0.002) ([Supplementary-material supplementary-material-1]), suggesting that the IR expansion and contraction may be a critical factor underlying the size variation of cp genomes [69–72]. The shortest length of the cp genome of *R. chinensis* was mainly attributed to the loss of a long fragment (∼9,800 bp in length) at the IRb/LSC boundary, which contains four genes including *rpl*2, *rpl*23, *trn*I-CAU, and *ycf*2. Previous studies have shown that the loss of a small number of genes, or even all, of the IR is more common in conifer species from cupressophytes and Pinaceae [73, 74]. Nonetheless, similar cases were still reported for several angiosperms, such as *Erodium* L'Hér., *Geranium* L., *Monsonia* L. [75], *Trifolium* L. [76], and *Vicia* L. [77]. For those species, the loss of the complete or partial IR has shifted numerous genes, e.g., *rpl*2, *rpl*23, and *ndh*B, into the SC regions. Recent studies have revealed that most genes that moved from the IR into the SC exhibited higher synonymous substitution rates consistent with the SC genes, and IR localization is a critical factor underlying the reduced substitution rates in plant plastomes [78]. Among the six Anacardiaceae species, the longest length of the cp genome was observed for *A. occidentale*, which was associated with the size expansion of *ycf*2-*trn*L spacer (∼6,200 bp longer than other five species) in the IR regions. Based on experimental evidence, Rabah et al. [79] have shown that an intracellular gene transfer event of mitochondrial DNA into the plastome may have occurred <20 million years ago in a single clade of the genus *Anacardium*. This event led to an insertion of an ∼6,700 bp fragment between *ycf*2 and *trn*L-CAA, which is also responsible for the highly expanded IR of *A. occidentale*.

We compared the complete cp genome structure of *T. vernicifluum* with five species from different genera in Anacardiaceae. Our results indicated that the six Anacardiaceae cp genomes were relatively conserved (Figure 4), which is consistent with the slow rates of sequence and structural evolution of plant plastomes [78, 80, 81]. However, the synteny analysis still detected a large inversed segment (16,910 bp in length) in the LSC region of *M. indica*. Similar events have been reported for other species of Malvids, such as *Aquilaria sinensis* (∼16,000 bp in length, from *rpl*20 to *rbc*L genes) [82]. Previous studies have shown that tRNA activity may be a key factor triggering the inversion events [83]. We found that both the upper and lower flanks of the inversed segment identified in this study had tRNA genes, i.e., *trn*E-UUC and *trn*T-UGU at the upper flank and *trn*T-GGU and *trn*L-UAA at the lower flank. Furthermore, higher genetic variation and lower GC content of the flank regions could also promote gene rearrangements in plastid genomes [84].

The mVISTA analysis showed that intergenic spacers were more variable than genes (Figure 2). Numerous divergence hotspot regions (e.g., *trn*H-*psb*A, *trn*T-*trn*L, *atp*F-*atp*H, *ccs*A-*ndh*D, *pet*D-*rpo*A, and *trn*L-*trn*F) were identified through calculating and comparing the nucleotide diversity (Pi) values (Figure 3). Those highly variable loci can be used as potential molecular markers for the phylogenetic studies in the future [85, 86]. Among those, *trn*L-*trn*F has been proved to be a useful marker for the phylogenetic studies of *Toxicodendron*. A total of 82 variable sites and 45 parsimony-informative sites were detected across 68 accessions of *Toxicodendron* and some closely related taxa [28]. A more detailed study identified 180 variable sites among the *trn*L-*trn*F sequences of 85 taxa, representing 22 of the 30 species in *Toxicodendron* and 60 related taxa in Anacardiaceae [87]. This marker was also found to be useful in analyzing the intraspecific variation of *T. vernicifluum*. Combined with the sequence of *trn*L intron, Suzuki et al. [3] have detected three chloroplast DNA haplotypes across populations from China, South Korea, and Japan. Notably, only one of them was shared by populations in northeastern China, South Korea, and Japan, suggesting that the Chinese lacquer trees in Japan were more likely to be introduced from Liaoning and Shandong provinces of China.

The phylogenetic relationships among several families of Sapindales, such as Sapindaceae, Simaroubaceae, Rutaceae, and Meliaceae, are not fully resolved in the Angiosperm Phylogeny Website (http://www.mobot.org/MOBOT/research/APweb/). A recent phylogenetic study based on plastid *rbc*L, *atp*B, and *trn*L-*trn*F sequences indicated that Simaroubaceae was sister to Meliaceae, with moderate support (0.98 PP/82 BS), but the position of Sapindaceae could not be resolved with confidence [30]. Using 80 genes of plastomes, Lin et al. [31] found that Simaroubaceae was sister to Rutaceae with strong support (100 BS), but the position of Sapindaceae was still poorly supported (57 BS). In this study, both BI and ML analyses generated a well-resolved phylogeny of the six families of Sapindales. Two distinct clades were recognized: one consisting of Anacardiaceae and Burseraceae (1.0 PP/100 BS) and the other comprised of the remaining four families (0.998 PP/83 BS). In addition, our results strongly supported that Simaroubaceae and Rutaceae form a monophyletic group as sister to Meliaceae (1.0 PP/100 BS). These findings were consistent with the results of the angiosperm phylogeny based on five plastid and nuclear markers [32] and also with the topology of the most recent plastid phylogenomic angiosperm (PPA) tree [88]. Notably, all of those studies using different dataset presented short internodes (e.g., at the base of the Sapindaceae + Simaroubaceae + Rutaceae + Meliaceae clade, and at the base of the Simaroubaceae + Rutaceae + Meliaceae clade) connected by long branches, indicating that rapid radiation may have occurred among those families [89, 90].

Within the Anacardiaceae, both BI and ML analyses supported that *T. vernicifluum* was more closely related to *R. chinensis* and two *Pistacia* species. This is consistent with the phylogeny obtained through three nuclear DNA (ITS, ETS, and NIA-i3) and two chloroplast (*ndh*F and *trn*L-*trn*F) regions [87]. However, due to lack of cp genome data for congeneric species, we did not provide more details on the intrageneric relationships of *Toxicodendron*. More studies on the plastomes of those species are expected to provide new insight into the evolutionary history of *T. vernicifluum* and its siblings.

## 5. Conclusions

In this study, we assembled, annotated, and analyzed the cp genome of *T. vernicifluum*, an important commercial arbor species widely cultivated in East Asia for producing highly durable lacquer. Forty-nine mononucleotide microsatellites, one dinucleotide microsatellite, two complex microsatellites, and 49 long repeats were determined. Several hotspots (e.g., *trn*H-*psb*A, *trn*T-*trn*L, *atp*F-*atp*H, *ccs*A-*ndh*D, *pet*D-*rpo*A, and *trn*L-*trn*F) of intergeneric divergence were also identified. The unique inversion (in *M. indica*), insertion (in *A. occidentale*), and gene loss (in *R. chinensis*) events may provide informative markers for phylogenetic resolution among different genera in Anacardiaceae. Both BI and ML analyses revealed that the genus *Toxicodendron* is closely related to *Pistacia* and *Rhus*. The phylogenetic relationships of the six families in Sapindales were well resolved, strongly supporting the topology that the clade including Simaroubaceae and Rutaceae is sister to Meliaceae. The genomic resources presented in this study will be useful for further studies on evolutionary patterns of *T. vernicifluum* and its closely related species.

## Figures and Tables

**Table 1:** Summary of features of six Anacardiaceae chloroplast genomes.

Genome features	*Toxicodendron vernicifluum*	*Rhus chinensis*	*Pistacia weinmannifolia*	*Anacardium occidentale*	*Mangifera indica*	*Spondias mombin*
Cp length	159,571	149,011	160,767	172,199	157,780	162,302
LSC length	87,475	96,882	88,402	87,727	86,673	89,938
SSC length	19,074	18,647	19,129	19,046	18,349	18,012
IR length	26,511	16,741	26,618	32,713	26,379	27,176
*ycf*2-*trn*L length	1,010	1,083	1,011	7,251	1,011	1,018
Genes	126	126	132	129	128	130
CDS	81	82	87	84	83	86
tRNA	37	36	37	37	37	36
rRNA	8	8	8	8	8	8
GC%	37.96	37.79	37.84	38.12	37.89	37.60

**Table 2:** Base composition of the *Toxicodendron vernicifluum* chloroplast genome.

Region	A (%)	T (U) (%)	C (%)	G (%)	AT (%)	GC (%)
LSC	31.33	32.59	18.53	17.55	63.92	36.08
SSC	33.83	33.54	16.97	15.67	67.37	32.63
IR	28.51	28.51	21.48	21.48	57.04	42.96
Total	30.74	31.31	19.06	18.90	62.04	37.96

## Data Availability

The complete chloroplast genome sequence can be found in GenBank with accession no. MK419151. The data used to support the findings of this study are included within the supplementary information files.

## Supplementary Materials

Supplementary Materials Figure S1: gene map and MAUVE alignment of five Anacardiaceae chloroplast genomes with *Rhus chinensis* removed. Figure S2: the linear correlation between the length of IR and the total length of the complete chloroplast genome sequence. Figure S3: the final alignment produced by the HomBlocks pipeline. Figure S4: visualization of genes that were integrated into the final alignment and their corresponding regions. Table S1: GenBank accession numbers of the complete chloroplast genome sequences of 52 species in Sapindales and two outgroups from Brassicales and Huerteales used for the phylogenetic analyses. Table S2: the best-fit partitioning schemes and DNA substitution models determined by PartitionFinder. Table S3: genes contained in the *Toxicodendron vernicifluum* chloroplast genome. Table S4: genes with introns in the *Toxicodendron vernicifluum* chloroplast genome. Table S5: the codon number and relative synonymous codon usage (RSCU) values calculated based on the coding sequences of 81 protein-coding genes in the complete chloroplast genome of *Toxicodendron vernicifluum*. Table S6: simple sequence repeats (SSRs) of the *Toxicodendron vernicifluum* chloroplast genome. Table S7: long repeats in the *Toxicodendron vernicifluum* chloroplast genome. Table S8: two single nucleotide variants between the complete chloroplast genome of *Toxicodendron vernicifluum* and *T. vernicifluum* cv. *Dahongpao*.
