# Supplementary material for: Complete Chloroplast Genome Sequence of Chinese Lacquer Tree (Toxicodendron vernicifluum, Anacardiaceae) and Its Phylogenetic Significance
Source: Biomed Res Int. 2020 Jan 30;2020:9014873. doi: 10.1155/2020/9014873 (PMC7011389; doi:10.1155/2020/9014873)
Supplement: Supplementary Materials — Figure S1: gene map and MAUVE alignment of five Anacardiaceae chloroplast genomes with Rhus chinensis removed. Figure S2: the linear correlation between the length of IR and the total length of the complete chloroplast genome sequence. Figure S3: the final alignment produced by the HomBlocks pipeline. Figure S4: visualization of genes that were integrated into the final alignment and their corresponding regions. Table S1: GenBank accession numbers of the complete chloroplast genome sequences of 52 species in Sapindales and two outgroups from Brassicales and Huerteales used for the phylogenetic analyses. Table S2: the best-fit partitioning schemes and DNA substitution models determined by PartitionFinder. Table S3: genes contained in the Toxicodendron vernicifluum chloroplast genome. Table S4: genes with introns in the Toxicodendron vernicifluum chloroplast genome. Table S5: the codon number and relative synonymous codon usage (RSCU) values calculated based on the coding sequences of 81 protein-coding genes in the complete chloroplast genome of Toxicodendron vernicifluum. Table S6: simple sequence repeats (SSRs) of the Toxicodendron vernicifluum chloroplast genome. Table S7: long repeats in the Toxicodendron vernicifluum chloroplast genome. Table S8: two single nucleotide variants between the complete chloroplast genome of Toxicodendron vernicifluum and T. vernicifluum cv. Dahongpao. [file 9014873.f1.zip › 9014873.f1/TableS1.docx]

**Table S1** Genbank accession numbers of the complete chloroplast genome sequences of 52 species in Sapindales and two outgroups from Brassicales and Huerteales used for the phylogenetic analyses.

| Order | Family | Genus | Species | Genbank accession number |
| --- | --- | --- | --- | --- |
| Brassicales | Caricaceae | *Carica* L. | *Carica papaya* L. | EU431223 |
| Huerteales | Tapisciaceae | *Tapiscia* Oliv. | *Tapiscia sinensis* Oliv. | MF926267 |
| Sapindales | Burseraceae | *Commiphora* Jacq. | *Commiphora gileadensis* (L.) C. Chr. | MH042752 |
| Sapindales | Burseraceae | *Commiphora* Jacq. | *Commiphora foliacea* Sprague | MH041484 |
| Sapindales | Burseraceae | *Commiphora* Jacq. | *Commiphora wightii* (Arn.) Bhandari | MF957201 |
| Sapindales | Burseraceae | *Boswellia* Roxb. ex Colebr. | *Boswellia sacra* Flueck. | KT934315 |
| Sapindales | Simaroubaceae | *Ailanthus* Desf. | *Ailanthus altissima* (Mill.) Swingle | MG799542 |
| Sapindales | Simaroubaceae | *Leitneria* Chapm. | *Leitneria floridana* Chapm. | KT692940 |
| Sapindales | Meliaceae | *Khaya* A. Juss. | *Khaya senegalensis* (Desr.) A. Juss. | KX364458 |
| Sapindales | Meliaceae | *Carapa* Aubl. | *Carapa guianensis* Aubl. | MH396436 |
| Sapindales | Meliaceae | *Xylocarpus* J. Koenig | *Xylocarpus rumphii* (Kostel.) Mabb. | MH330687 |
| Sapindales | Meliaceae | *Xylocarpus* J. Koenig | *Xylocarpus granatum* J. Koenig | MH348155 |
| Sapindales | Meliaceae | *Xylocarpus* J. Koenig | *Xylocarpus moluccensis* (Lam.) M. Roem. | MH330688 |
| Sapindales | Meliaceae | *Swietenia* Jacq. | *Swietenia mahagoni* (L.) Jacq. | MH348156 |
| Sapindales | Meliaceae | *Entandrophragma* C. DC. | *Entandrophragma cylindricum* (Sprague) Sprague | KY923074 |
| Sapindales | Meliaceae | *Toona* (Endl.) M. Roem. | *Toona ciliata* M. Roem. | MG813875 |
| Sapindales | Meliaceae | *Cedrela* P. Browne | *Cedrela odorata* L. | MG724915 |
| Sapindales | Meliaceae | *Azadirachta* A. Juss. | *Azadirachta indica* A. Juss. | KF986530 |
| Sapindales | Anacardiaceae | *Spondias* L. | *Spondias tuberosa* Arruda | KU756562 |
| Sapindales | Anacardiaceae | *Spondias* L. | *Spondias mombin* L. | KY828469 |
| Sapindales | Anacardiaceae | *Spondias* L. | *Spondias bahiensis* P.Carvalho, Van den Berg & M.Machado | KU756561 |
| Sapindales | Anacardiaceae | *Pistacia* L. | *Pistacia vera* L. | KY549635 |
| Sapindales | Anacardiaceae | *Pistacia* L. | *Pistacia weinmaniifolia* J. Poiss. ex Franch. | MF630953 |
| Sapindales | Anacardiaceae | *Mangifera* L. | *Mangifera indica* L. | KY635882 |
| Sapindales | Anacardiaceae | *Toxicodendron* Mill. | *Toxicodendron vernicifluum* (Stokes) F. A. Barkley | MK419151 |
| Sapindales | Anacardiaceae | *Rhus* L. | *Rhus chinensis* Mill. | KX447140 |
| Sapindales | Anacardiaceae | *Anacardium* L. | *Anacardium occidentale* L. | KY635877 |
| Sapindales | Sapindaceae | *Dodonaea* Mill. | *Dodonaea viscosa* Jacq. | MF155892 |
| Sapindales | Sapindaceae | *Dipteronia* Oliv. | *Dipteronia dyeriana* Henry | KT985457 |
| Sapindales | Sapindaceae | *Dipteronia* Oliv. | *Dipteronia sinensis* Oliv. | KT878501 |
| Sapindales | Sapindaceae | *Litchi* Sonn. | *Litchi chinensis* Sonn. | KY635881 |
| Sapindales | Sapindaceae | *Dimocarpus* Lour. | *Dimocarpus longan* Lour. | MG214255 |
| Sapindales | Sapindaceae | *Koelreuteria* Laxm. | *Koelreuteria paniculata* Laxm. | KY859413 |
| Sapindales | Sapindaceae | *Aesculus* L. | *Aesculus wangii* Hu | MF583747 |
| Sapindales | Sapindaceae | *Acer* L. | *Acer buergerianum* Miq. | KY419137 |
| Sapindales | Sapindaceae | *Acer* L. | *Acer sino-oblongum* Metc. | KY987160 |
| Sapindales | Sapindaceae | *Acer* L. | *Acer morrisonense* Hayata | [KT970611](https://www.ncbi.nlm.nih.gov/nuccore/KT970611) |
| Sapindales | Sapindaceae | *Eurycorymbus* Hand.-Mazz. | *Eurycorymbus cavaleriei* (H. Lév.) Rehder & Hand.-Mazz. | MG813997 |
| Sapindales | Sapindaceae | *Xanthoceras* Bunge | *Xanthoceras sorbifolium* Bunge | KY779850 |
| Sapindales | Sapindaceae | *Sapindus* L. | *Sapindus mukorossi* Gaertn. | KM454982 |
| Sapindales | Rutaceae | *Citrus* L. | *Citrus aurantiifolia* (Christm.) Swingle | KJ865401 |
| Sapindales | Rutaceae | *Citrus* L. | *Citrus sinensis* (L.) Osbeck | DQ864733 |
| Sapindales | Rutaceae | *Citrus* L. | *Citrus platymamma* hort. ex Tanaka | KR259987 |
| Sapindales | Rutaceae | *Zanthoxylum* L. | *Zanthoxylum piperitum* DC. | KT153018 |
| Sapindales | Rutaceae | *Zanthoxylum* L. | *Zanthoxylum schinifolium* Sieb. & Zucc. | KT321318 |
| Sapindales | Rutaceae | *Zanthoxylum* L. | *Zanthoxylum simulans* Hance | MF716524 |
| Sapindales | Rutaceae | *Zanthoxylum* L. | *Zanthoxylum bungeanum* Maxim. | KX497031 |
| Sapindales | Rutaceae | *Phellodendron* Rupr. | *Phellodendron amurense* Rupr. | KY707335 |
| Sapindales | Rutaceae | *Clausena* Burm. f. | *Clausena excavata* Burm. f. | KU949003 |
| Sapindales | Rutaceae | *Murraya* J. Koenig | *Murraya koenigii* (L.) Spreng. | KU949002 |
| Sapindales | Rutaceae | *Atalantia* Corrêa | *Atalantia kwangtungensis* Merr. | MH329190 |
| Sapindales | Rutaceae | *Merrillia* Swingle | *Merrillia caloxylon* (Ridl.) Swingle | KU949006 |
| Sapindales | Rutaceae | *Glycosmis* Corrêa | *Glycosmis pentaphylla* (Retz.) DC. | KU949005 |
| Sapindales | Rutaceae | *Micromelum* Blume | *Micromelum minutum* Wight & Arn. | KU949007 |
