# Supplementary material for: Complete Chloroplast Genome Sequence of Chinese Lacquer Tree (Toxicodendron vernicifluum, Anacardiaceae) and Its Phylogenetic Significance
Source: Biomed Res Int. 2020 Jan 30;2020:9014873. doi: 10.1155/2020/9014873 (PMC7011389; doi:10.1155/2020/9014873)
Supplement: Supplementary Materials — Figure S1: gene map and MAUVE alignment of five Anacardiaceae chloroplast genomes with Rhus chinensis removed. Figure S2: the linear correlation between the length of IR and the total length of the complete chloroplast genome sequence. Figure S3: the final alignment produced by the HomBlocks pipeline. Figure S4: visualization of genes that were integrated into the final alignment and their corresponding regions. Table S1: GenBank accession numbers of the complete chloroplast genome sequences of 52 species in Sapindales and two outgroups from Brassicales and Huerteales used for the phylogenetic analyses. Table S2: the best-fit partitioning schemes and DNA substitution models determined by PartitionFinder. Table S3: genes contained in the Toxicodendron vernicifluum chloroplast genome. Table S4: genes with introns in the Toxicodendron vernicifluum chloroplast genome. Table S5: the codon number and relative synonymous codon usage (RSCU) values calculated based on the coding sequences of 81 protein-coding genes in the complete chloroplast genome of Toxicodendron vernicifluum. Table S6: simple sequence repeats (SSRs) of the Toxicodendron vernicifluum chloroplast genome. Table S7: long repeats in the Toxicodendron vernicifluum chloroplast genome. Table S8: two single nucleotide variants between the complete chloroplast genome of Toxicodendron vernicifluum and T. vernicifluum cv. Dahongpao. [file 9014873.f1.zip › 9014873.f1/TableS3.docx]

**Table S3** Genes contained in the *Toxicodendron vernicifluum* chloroplast genome.

| Category | Group of gene | Name of gene | | | |
| --- | --- | --- | --- | --- | --- |
| Self-replication | Ribosomal RNA genes | *rrn*4.5^a^ | *rrn*5^a^ | *rrn*16^a^ | *rrn*23^a^ |
|  | Transfer RNA genes | *trn*A-UGC^a^* | *trn*C-GCA | *trn*D-GUC | *trn*E-UUC |
|  |  | *trn*F-GAA | *trn*fM-CAU | *trn*G-GCC | *trn*H-GUG |
|  |  | *trn*I-CAU^a^ | *trn*I-GAU^a^* | *trn*K-UUU* | *trn*L-CAA^a^ |
|  |  | *trn*L-UAA* | *trn*L-UAG | *trn*M-CAU | *trn*N-GUU^a^ |
|  |  | *trn*P-UGG | *trn*Q-UUG | *trn*R-ACG^a^ | *trn*R-UCU |
|  |  | *trn*S-GCU | *trn*S-GGA | *trn*S-UGA | *trn*T-CGU* |
|  |  | *trn*T-GGU | *trn*T-UGU | *trn*V-GAC^a^ | *trn*V-UAC* |
|  |  | *trn*W-CCA | *trn*Y-GUA |  |  |
|  | Small subunit of ribosome | *rps*2 | *rps*3 | *rps*4 | *rps*7^a^ |
|  |  | *rps*8 | *rps*11 | *rps*12^a^ | *rps*14 |
|  |  | *rps*15 | *rps*16* | *rps*18 | *rps*19 |
|  | Large subunit of ribosome | *rpl*2^a^* | *rpl*14 | *rpl*16 | *rpl*20 |
|  |  | *rpl*22 | *rpl*23^a^ | *rpl*32 | *rpl*33 |
|  |  | *rpl*36 |  |  |  |
|  | RNA polymerase subunits | *rpo*A | *rpo*B | *rpo*C1* | *rpo*C2 |
| Photosynthesis | Subunits of photosystem I | *psa*A | *psa*B | *psa*C | *psa*I |
|  |  | *psa*J |  |  |  |
|  | Subunits of photosystem II | *psb*A | *psb*B | *psb*C | *psb*D |
|  |  | *psb*E | *psb*F | *psb*H | *psb*I |
|  |  | *psb*J | *psb*K | *psb*M | *psb*Z |
|  | Subunits of cytochrome | *pet*A | *pet*B | *pet*D |  |
|  | Subunits of ATP synthase | *atp*A | *atp*B | *atp*E | *atp*F* |
|  |  | *atp*H | *atp*I |  |  |
|  | Large subunit of Rubisco | *rbc*L |  |  |  |
|  | Subunits of NADH dehydrogenase | *ndh*A* | *ndh*B^a^* | *ndh*C | *ndh*D |
|  |  | *ndh*E | *ndh*F | *ndh*G | *ndh*H |
|  |  | *ndh*I | *ndh*J | *ndh*K |  |
| Other gene | Maturase | *mat*K |  |  |  |
|  | Envelope membrane protein | *cem*A |  |  |  |
|  | Subunit of acetyl-CoA | *acc*D |  |  |  |
|  | C-type cytochrome synthesis gene | *ccs*A |  |  |  |
|  | Protease | *clp*P** |  |  |  |
| Unknown function | Conserved open reading frames | *ycf*1^a^ | *ycf*2^a^ | *ycf*3** | *ycf*4 |
|  |  | *ycf*68^a^ |  |  |  |

a, Two gene copies in IRs; *, one-intron containing genes, **, two-intron containing genes.
