# Supplementary material for: Complete Chloroplast Genome Sequence of Chinese Lacquer Tree (Toxicodendron vernicifluum, Anacardiaceae) and Its Phylogenetic Significance
Source: Biomed Res Int. 2020 Jan 30;2020:9014873. doi: 10.1155/2020/9014873 (PMC7011389; doi:10.1155/2020/9014873)
Supplement: Supplementary Materials — Figure S1: gene map and MAUVE alignment of five Anacardiaceae chloroplast genomes with Rhus chinensis removed. Figure S2: the linear correlation between the length of IR and the total length of the complete chloroplast genome sequence. Figure S3: the final alignment produced by the HomBlocks pipeline. Figure S4: visualization of genes that were integrated into the final alignment and their corresponding regions. Table S1: GenBank accession numbers of the complete chloroplast genome sequences of 52 species in Sapindales and two outgroups from Brassicales and Huerteales used for the phylogenetic analyses. Table S2: the best-fit partitioning schemes and DNA substitution models determined by PartitionFinder. Table S3: genes contained in the Toxicodendron vernicifluum chloroplast genome. Table S4: genes with introns in the Toxicodendron vernicifluum chloroplast genome. Table S5: the codon number and relative synonymous codon usage (RSCU) values calculated based on the coding sequences of 81 protein-coding genes in the complete chloroplast genome of Toxicodendron vernicifluum. Table S6: simple sequence repeats (SSRs) of the Toxicodendron vernicifluum chloroplast genome. Table S7: long repeats in the Toxicodendron vernicifluum chloroplast genome. Table S8: two single nucleotide variants between the complete chloroplast genome of Toxicodendron vernicifluum and T. vernicifluum cv. Dahongpao. [file 9014873.f1.zip › 9014873.f1/TableS4.docx]

**Table S4** Genes with introns in the *Toxicodendron vernicifluum* chloroplast genome.

| Gene | Location | Exon I  (bp) | Exon II  (bp) | Exon III  (bp) | Intron I  (bp) | Intron II  (bp) |
| --- | --- | --- | --- | --- | --- | --- |
| *trn*K-UUU | LSC | 38 | 36 |  | 2,592 |  |
| *rps*16 | LSC | 40 | 224 |  | 878 |  |
| *trn*T-CGU | LSC | 34 | 44 |  | 710 |  |
| *atp*F | LSC | 161 | 406 |  | 743 |  |
| *rpo*C1 | LSC | 453 | 1,611 |  | 780 |  |
| *ycf*3 | LSC | 124 | 230 | 153 | 733 | 800 |
| *trn*L-UAA | LSC | 35 | 50 |  | 426 |  |
| *trn*V-UAC | LSC | 38 | 57 |  | 567 |  |
| *clp*P | LSC | 71 | 287 | 230 | 829 | 640 |
| *rpl*2* | IRa, IRb | 391 | 470 |  | 629 |  |
| *ndh*B* | IRa, IRb | 775 | 758 |  | 681 |  |
| *trn*I-GAU* | IRb, IRb | 33 | 41 |  | 958 |  |
| *trn*A-UGC* | IRa, IRb | 37 | 36 |  | 841 |  |
| *ndh*A | SSC | 553 | 539 |  | 1,116 |  |

*, Two gene copies in IRs.
