# Supplementary material for: Complete Chloroplast Genome Sequence of Chinese Lacquer Tree (Toxicodendron vernicifluum, Anacardiaceae) and Its Phylogenetic Significance
Source: Biomed Res Int. 2020 Jan 30;2020:9014873. doi: 10.1155/2020/9014873 (PMC7011389; doi:10.1155/2020/9014873)
Supplement: Supplementary Materials — Figure S1: gene map and MAUVE alignment of five Anacardiaceae chloroplast genomes with Rhus chinensis removed. Figure S2: the linear correlation between the length of IR and the total length of the complete chloroplast genome sequence. Figure S3: the final alignment produced by the HomBlocks pipeline. Figure S4: visualization of genes that were integrated into the final alignment and their corresponding regions. Table S1: GenBank accession numbers of the complete chloroplast genome sequences of 52 species in Sapindales and two outgroups from Brassicales and Huerteales used for the phylogenetic analyses. Table S2: the best-fit partitioning schemes and DNA substitution models determined by PartitionFinder. Table S3: genes contained in the Toxicodendron vernicifluum chloroplast genome. Table S4: genes with introns in the Toxicodendron vernicifluum chloroplast genome. Table S5: the codon number and relative synonymous codon usage (RSCU) values calculated based on the coding sequences of 81 protein-coding genes in the complete chloroplast genome of Toxicodendron vernicifluum. Table S6: simple sequence repeats (SSRs) of the Toxicodendron vernicifluum chloroplast genome. Table S7: long repeats in the Toxicodendron vernicifluum chloroplast genome. Table S8: two single nucleotide variants between the complete chloroplast genome of Toxicodendron vernicifluum and T. vernicifluum cv. Dahongpao. [file 9014873.f1.zip › 9014873.f1/TableS5.docx]

**Table S5** The codon number and relative synonymous codon usage (RSCU) values calculated based on the coding sequences of 81 protein-coding genes in the complete chloroplast genome of *Toxicodendron vernicifluum*.

| Amino acid | Codon | Number | RSCU | Amino acid | Codon | Number | RSCU |
| --- | --- | --- | --- | --- | --- | --- | --- |
| Ala | GCA | 386 | 1.09 | Ile | AUA | 673 | 0.90 |
| Ala | GCC | 225 | 0.64 | Ile | AUC | 480 | 0.64 |
| Ala | GCG | 182 | 0.52 | Ile | AUU | 1096 | 1.46 |
| Ala | GCU | 620 | 1.76 | Lys | AAA | 1047 | 1.47 |
| Arg | AGA | 479 | 1.78 | Lys | AAG | 381 | 0.53 |
| Arg | AGG | 185 | 0.69 | Met | AUG | 617 | 1.00 |
| Arg | CGA | 366 | 1.36 | Ple | UUC | 568 | 0.73 |
| Arg | CGC | 119 | 0.44 | Ple | UUU | 979 | 1.27 |
| Arg | CGG | 142 | 0.53 | Pro | CCA | 311 | 1.13 |
| Arg | CGU | 322 | 1.20 | Pro | CCC | 205 | 0.75 |
| Asn | AAC | 315 | 0.48 | Pro | CCG | 164 | 0.60 |
| Asn | AAU | 995 | 1.52 | Pro | CCU | 419 | 1.53 |
| Asp | GAC | 239 | 0.43 | Ser | AGC | 132 | 0.38 |
| Asp | GAU | 879 | 1.57 | Ser | AGU | 418 | 1.19 |
| Cys | UGC | 84 | 0.53 | Ser | UCA | 417 | 1.19 |
| Cys | UGU | 234 | 1.47 | Ser | UCC | 363 | 1.03 |
| Gln | CAA | 709 | 1.53 | Ser | UCG | 215 | 0.61 |
| Gln | CAG | 220 | 0.47 | Ser | UCU | 562 | 1.60 |
| Glu | GAA | 1073 | 1.49 | Ter | UAA | 45 | 1.67 |
| Glu | GAG | 369 | 0.51 | Ter | UAG | 22 | 0.81 |
| Gly | GGA | 733 | 1.61 | Ter | UGA | 14 | 0.52 |
| Gly | GGC | 173 | 0.38 | Thr | ACA | 404 | 1.20 |
| Gly | GGG | 328 | 0.72 | Thr | ACC | 256 | 0.76 |
| Gly | GGU | 589 | 1.29 | Thr | ACG | 161 | 0.48 |
| His | CAC | 177 | 0.54 | Thr | ACU | 526 | 1.56 |
| His | CAU | 480 | 1.46 | Trp | UGG | 464 | 1.00 |
| Leu | CUA | 406 | 0.87 | Tyr | UAC | 194 | 0.40 |
| Leu | CUC | 207 | 0.44 | Tyr | UAU | 780 | 1.60 |
| Leu | CUG | 208 | 0.45 | Val | GUA | 533 | 1.49 |
| Leu | CUU | 572 | 1.23 | Val | GUC | 181 | 0.51 |
| Leu | UUA | 814 | 1.75 | Val | GUG | 205 | 0.57 |
| Leu | UUG | 587 | 1.26 | Val | GUU | 511 | 1.43 |
