# Supplementary material for: Complete Chloroplast Genome Sequence of Chinese Lacquer Tree (Toxicodendron vernicifluum, Anacardiaceae) and Its Phylogenetic Significance
Source: Biomed Res Int. 2020 Jan 30;2020:9014873. doi: 10.1155/2020/9014873 (PMC7011389; doi:10.1155/2020/9014873)
Supplement: Supplementary Materials — Figure S1: gene map and MAUVE alignment of five Anacardiaceae chloroplast genomes with Rhus chinensis removed. Figure S2: the linear correlation between the length of IR and the total length of the complete chloroplast genome sequence. Figure S3: the final alignment produced by the HomBlocks pipeline. Figure S4: visualization of genes that were integrated into the final alignment and their corresponding regions. Table S1: GenBank accession numbers of the complete chloroplast genome sequences of 52 species in Sapindales and two outgroups from Brassicales and Huerteales used for the phylogenetic analyses. Table S2: the best-fit partitioning schemes and DNA substitution models determined by PartitionFinder. Table S3: genes contained in the Toxicodendron vernicifluum chloroplast genome. Table S4: genes with introns in the Toxicodendron vernicifluum chloroplast genome. Table S5: the codon number and relative synonymous codon usage (RSCU) values calculated based on the coding sequences of 81 protein-coding genes in the complete chloroplast genome of Toxicodendron vernicifluum. Table S6: simple sequence repeats (SSRs) of the Toxicodendron vernicifluum chloroplast genome. Table S7: long repeats in the Toxicodendron vernicifluum chloroplast genome. Table S8: two single nucleotide variants between the complete chloroplast genome of Toxicodendron vernicifluum and T. vernicifluum cv. Dahongpao. [file 9014873.f1.zip › 9014873.f1/TableS6.docx]

**Table S6** Simple sequence repeats (SSRs) of the *Toxicodendron vernicifluum* chloroplast genome.

| ID | SSR type | Repeat Motif | Length (bp) | Start | End | Region | Location |
| --- | --- | --- | --- | --- | --- | --- | --- |
| 1 | p1 | (A)10 | 10 | 3,917 | 3,926 | LSC | IGS |
| 2 | p1 | (A)10 | 10 | 5,295 | 5,304 | LSC | IGS |
| 3 | p1 | (A)11 | 11 | 9,945 | 9,955 | LSC | IGS |
| 4 | p1 | (A)13 | 13 | 14,583 | 14,595 | LSC | IGS |
| 5 | p1 | (T)14 | 14 | 14,719 | 14,732 | LSC | IGS |
| 6 | p1 | (T)11 | 11 | 17,839 | 17,849 | LSC | IGS |
| 7 | p1 | (T)11 | 11 | 20,085 | 20,095 | LSC | *rpo*C2 |
| 8 | p1 | (A)12 | 12 | 30,500 | 30,511 | LSC | IGS |
| 9 | p1 | (T)11 | 11 | 31,142 | 31,152 | LSC | IGS |
| 10 | p1 | (A)10 | 10 | 32,213 | 32,222 | LSC | IGS |
| 11 | p1 | (T)10 | 10 | 33,499 | 33,508 | LSC | IGS |
| 12 | p2 | (AT)6 | 12 | 33,825 | 33,836 | LSC | IGS |
| 13 | p1 | (A)11 | 11 | 38,446 | 38,456 | LSC | IGS |
| 14 | p1 | (A)10 | 10 | 38,980 | 38,989 | LSC | IGS |
| 15 | p1 | (T)10 | 10 | 45,946 | 45,955 | LSC | *ycf*3*** |
| 16 | c | (A)10gaccccgatcggttgattcgttccaattcattgattgaatccggtataaatatcag(A)12 | 78 | 47,214 | 47,291 | LSC | IGS |
| 17 | p1 | (A)10 | 10 | 49,648 | 49,657 | LSC | IGS |
| 18 | p1 | (T)11 | 11 | 51,399 | 51,409 | LSC | IGS |
| 19 | p1 | (T)10 | 10 | 56,771 | 56,780 | LSC | *atp*B |
| 20 | p1 | (A)10 | 10 | 57,353 | 57,362 | LSC | IGS |
| 21 | p1 | (T)10 | 10 | 61,163 | 61,172 | LSC | IGS |
| 22 | p1 | (T)11 | 11 | 61,286 | 6,1296 | LSC | IGS |
| 23 | p1 | (T)10 | 10 | 62,015 | 62,024 | LSC | IGS |
| 24 | p1 | (A)11 | 11 | 62,373 | 62,383 | LSC | IGS |
| 25 | p1 | (T)11 | 11 | 63,299 | 63,309 | LSC | IGS |
| 26 | p1 | (T)12 | 12 | 63,563 | 63,574 | LSC | IGS |
| 27 | p1 | (G)11 | 11 | 63,682 | 63,692 | LSC | IGS |
| 28 | p1 | (T)11 | 11 | 66,276 | 66,286 | LSC | IGS |
| 29 | p1 | (A)11 | 11 | 66,449 | 66,459 | LSC | IGS |
| 30 | p1 | (T)11 | 11 | 67,943 | 67,953 | LSC | IGS |
| 31 | p1 | (G)11 | 11 | 69,290 | 69,300 | LSC | IGS |
| 32 | c | (G)10attc(T)10 | 24 | 69,487 | 69,510 | LSC | IGS |
| 33 | p1 | (T)14 | 14 | 70,256 | 70,269 | LSC | IGS |
| 34 | p1 | (A)12 | 12 | 73,709 | 73,720 | LSC | *clp*P*** |
| 35 | p1 | (A)14 | 14 | 84,294 | 84,307 | LSC | IGS |
| 36 | p1 | (T)10 | 10 | 85,030 | 85,039 | LSC | IGS |
| 37 | p1 | (T)10 | 10 | 87,488 | 87,497 | LSC | IGS |
| 38 | p1 | (C)10 | 10 | 101,444 | 101,453 | IRa | IGS |
| 39 | p1 | (T)12 | 12 | 102,517 | 102,528 | IRa | IGS |
| 40 | p1 | (T)10 | 10 | 111,975 | 111,984 | IRa | IGS |
| 41 | p1 | (A)12 | 12 | 113,835 | 113,846 | IRa | *ycf*1 |
| 42 | p1 | (T)12 | 12 | 116,760 | 116,771 | SSC | IGS |
| 43 | p1 | (A)14 | 14 | 117,637 | 117,650 | SSC | IGS |
| 44 | p1 | (T)13 | 13 | 117,815 | 117,827 | SSC | IGS |
| 45 | p1 | (T)10 | 10 | 123,475 | 123,484 | SSC | IGS |
| 46 | p1 | (T)10 | 10 | 131,290 | 131,299 | IRb | *ycf*1 |
| 47 | p1 | (A)10 | 10 | 132,925 | 132,934 | IRb | *ycf*1 |
| 48 | p1 | (T)12 | 12 | 133,201 | 133,212 | IRb | *ycf*1 |
| 49 | p1 | (A)10 | 10 | 135,063 | 135,072 | IRb | IGS |
| 50 | p1 | (A)12 | 12 | 144,519 | 144,530 | IRb | IGS |
| 51 | p1 | (G)10 | 10 | 145,594 | 145,603 | IRb | IGS |
| 52 | p1 | (A)10 | 10 | 159,550 | 159,559 | IRb | IGS |

P1, mononucleotide SSR; P2, dinucleotide SSR; C, complex SSR; IGS, the SSR is located in the intergenic region; *, the SSR is located in the intron of the gene.
