# Supplementary material for: Complete Chloroplast Genome Sequence of Chinese Lacquer Tree (Toxicodendron vernicifluum, Anacardiaceae) and Its Phylogenetic Significance
Source: Biomed Res Int. 2020 Jan 30;2020:9014873. doi: 10.1155/2020/9014873 (PMC7011389; doi:10.1155/2020/9014873)
Supplement: Supplementary Materials — Figure S1: gene map and MAUVE alignment of five Anacardiaceae chloroplast genomes with Rhus chinensis removed. Figure S2: the linear correlation between the length of IR and the total length of the complete chloroplast genome sequence. Figure S3: the final alignment produced by the HomBlocks pipeline. Figure S4: visualization of genes that were integrated into the final alignment and their corresponding regions. Table S1: GenBank accession numbers of the complete chloroplast genome sequences of 52 species in Sapindales and two outgroups from Brassicales and Huerteales used for the phylogenetic analyses. Table S2: the best-fit partitioning schemes and DNA substitution models determined by PartitionFinder. Table S3: genes contained in the Toxicodendron vernicifluum chloroplast genome. Table S4: genes with introns in the Toxicodendron vernicifluum chloroplast genome. Table S5: the codon number and relative synonymous codon usage (RSCU) values calculated based on the coding sequences of 81 protein-coding genes in the complete chloroplast genome of Toxicodendron vernicifluum. Table S6: simple sequence repeats (SSRs) of the Toxicodendron vernicifluum chloroplast genome. Table S7: long repeats in the Toxicodendron vernicifluum chloroplast genome. Table S8: two single nucleotide variants between the complete chloroplast genome of Toxicodendron vernicifluum and T. vernicifluum cv. Dahongpao. [file 9014873.f1.zip › 9014873.f1/TableS7.docx]

**Table S7** Long repeats in the *Toxicodendron vernicifluum* chloroplast genome.

| ID | Repeat Start 1 | Type | Size (bp) | Repeat Start 2 | Mismatch (bp) | E-Value | Gene | Region |
| --- | --- | --- | --- | --- | --- | --- | --- | --- |
| 1 | 94674 | F | 66 | 94692 | -1 | 2.6×10^-28^ | *ycf*2 | IRb |
| 2 | 94674 | P | 66 | 152288 | -1 | 2.6×10^-28^ | *ycf*2 | IRb; IRa |
| 3 | 94692 | P | 66 | 152306 | -1 | 2.6×10^-28^ | *ycf*2 | IRb; IRa |
| 4 | 152288 | F | 66 | 152306 | -1 | 2.6×10^-28^ | *ycf*2 | IRa |
| 5 | 94688 | F | 52 | 94706 | 0 | 3.53×10^-22^ | *ycf*2 | IRb |
| 6 | 94688 | P | 52 | 152288 | 0 | 3.53×10^-22^ | *ycf*2 | IRb; IRa |
| 7 | 94706 | P | 52 | 152306 | 0 | 3.53×10^-22^ | *ycf*2 | IRb; IRa |
| 8 | 94674 | F | 48 | 94710 | -1 | 1.3×10^-17^ | *ycf*2 | IRb |
| 9 | 94674 | P | 48 | 152288 | -1 | 1.3×10^-17^ | *ycf*2 | IRb; IRa |
| 10 | 94710 | P | 48 | 152324 | -1 | 1.3×10^-17^ | *ycf*2 | IRb; IRa |
| 11 | 152288 | F | 48 | 152324 | -1 | 1.3×10^-17^ | *ycf*2 | IRa |
| 12 | 30853 | P | 42 | 30853 | 0 | 3.7×10^-16^ | IGS | LSC |
| 13 | 41430 | F | 41 | 43654 | 0 | 1.48×10^-15^ | *psa*B; *psa*A | LSC |
| 14 | 6104 | P | 38 | 6104 | 0 | 9.48×10^-14^ | IGS | LSC |
| 15 | 102103 | F | 41 | 124948 | -1 | 1.82×10^-13^ | *rps*12;IGS | IRb; SSC |
| 16 | 124948 | P | 41 | 144902 | -1 | 1.82×10^-13^ | IGS;*rps*12 | SSC; IRa |
| 17 | 102108 | F | 36 | 124953 | 0 | 1.52×10^-12^ | *rps*12;IGS | IRb; SSC |
| 18 | 124953 | P | 36 | 144902 | 0 | 1.52×10^-12^ | IGS;*rps*12 | SSC; IRa |
| 19 | 94688 | F | 34 | 94724 | 0 | 2.43×10^-11^ | *ycf*2 | IRb |
| 20 | 94688 | P | 34 | 152288 | 0 | 2.43×10^-11^ | *ycf*2 | IRb; IRa |
| 21 | 94724 | P | 34 | 152324 | 0 | 2.43×10^-11^ | *ycf*2 | IRb; IRa |
| 22 | 305 | R | 31 | 305 | 0 | 1.55×10^-9^ | IGS | LSC |
| 23 | 9616 | P | 30 | 47569 | 0 | 6.21×10^-9^ | IGS; *trn*S*-*GGA | LSC |
| 24 | 48739 | F | 27 | 48766 | 0 | 3.98×10^-7^ | IGS | LSC |
| 25 | 94674 | F | 30 | 94728 | -1 | 5.59×10^-7^ | *ycf*2 | IRb |
| 26 | 94674 | P | 30 | 152288 | -1 | 5.59×10^-7^ | *ycf*2 | IRb; IRa |
| 27 | 94728 | P | 30 | 152342 | -1 | 5.59×10^-7^ | *ycf*2 | IRb; IRa |
| 28 | 111195 | F | 30 | 111226 | -1 | 5.59×10^-7^ | *rrn*4.5 | IRb |
| 29 | 111195 | P | 30 | 135790 | -1 | 5.59×10^-7^ | *rrn*4.5 | IRb; IRa |
| 30 | 111226 | P | 30 | 135821 | -1 | 5.59×10^-7^ | *rrn*4.5 | IRb; IRa |
| 31 | 135790 | F | 30 | 135821 | -1 | 5.59×10^-7^ | *rrn*4.5 | IRa |
| 32 | 152288 | F | 30 | 152342 | -1 | 5.59×10^-7^ | *ycf*2 | IRa |
| 33 | 299 | R | 25 | 299 | 0 | 6.36×10^-6^ | IGS | LSC |
| 34 | 44956 | F | 25 | 44968 | 0 | 6.36×10^-6^ | IGS | LSC |
| 35 | 19114 | F | 24 | 19135 | 0 | 2.54×10^-5^ | *rpo*C2 | LSC |
| 36 | 116704 | F | 23 | 116727 | 0 | 1.02×10^-4^ | IGS | SSC |
| 37 | 135797 | F | 23 | 135828 | 0 | 1.02×10^-4^ | *rrn*4.5 | IRa |
| 38 | 11207 | F | 26 | 39195 | -1 | 1.24×10^-4^ | *atp*A; *trn*G*-*GCC | LSC |
| 39 | 11262 | P | 22 | 11291 | 0 | 4.07×10^-4^ | IGS | LSC |
| 40 | 16340 | R | 22 | 16340 | 0 | 4.07×10^-4^ | *atp*I | LSC |
| 41 | 85541 | R | 22 | 85541 | 0 | 4.07×10^-4^ | IGS | LSC |
| 42 | 73203 | F | 25 | 73230 | -1 | 4.77×10^-4^ | IGS | LSC |
| 43 | 94113 | F | 25 | 94155 | -1 | 4.77×10^-4^ | *ycf*2 | IRb |
| 44 | 94113 | P | 25 | 152866 | -1 | 4.77×10^-4^ | *ycf*2 | IRb; IRa |
| 45 | 94155 | P | 25 | 152908 | -1 | 4.77×10^-4^ | *ycf*2 | IRb; IRa |
| 46 | 94662 | F | 25 | 94698 | -1 | 4.77×10^-4^ | *ycf*2 | IRb |
| 47 | 94662 | F | 25 | 94716 | -1 | 4.77×10^-4^ | *ycf*2 | IRb |
| 48 | 94662 | P | 25 | 152305 | -1 | 4.77×10^-4^ | *ycf*2 | IRb; IRa |
| 49 | 94662 | P | 25 | 152323 | -1 | 4.77×10^-4^ | *ycf*2 | IRb; IRa |

F: forward; P: palindromic; R: reverse; IGS: intergenic space.
