# Supplementary figures and images for: Complete Chloroplast Genome Sequence of Chinese Lacquer Tree (Toxicodendron vernicifluum, Anacardiaceae) and Its Phylogenetic Significance
Source: Biomed Res Int. 2020 Jan 30;2020:9014873. doi: 10.1155/2020/9014873 (PMC7011389; doi:10.1155/2020/9014873)

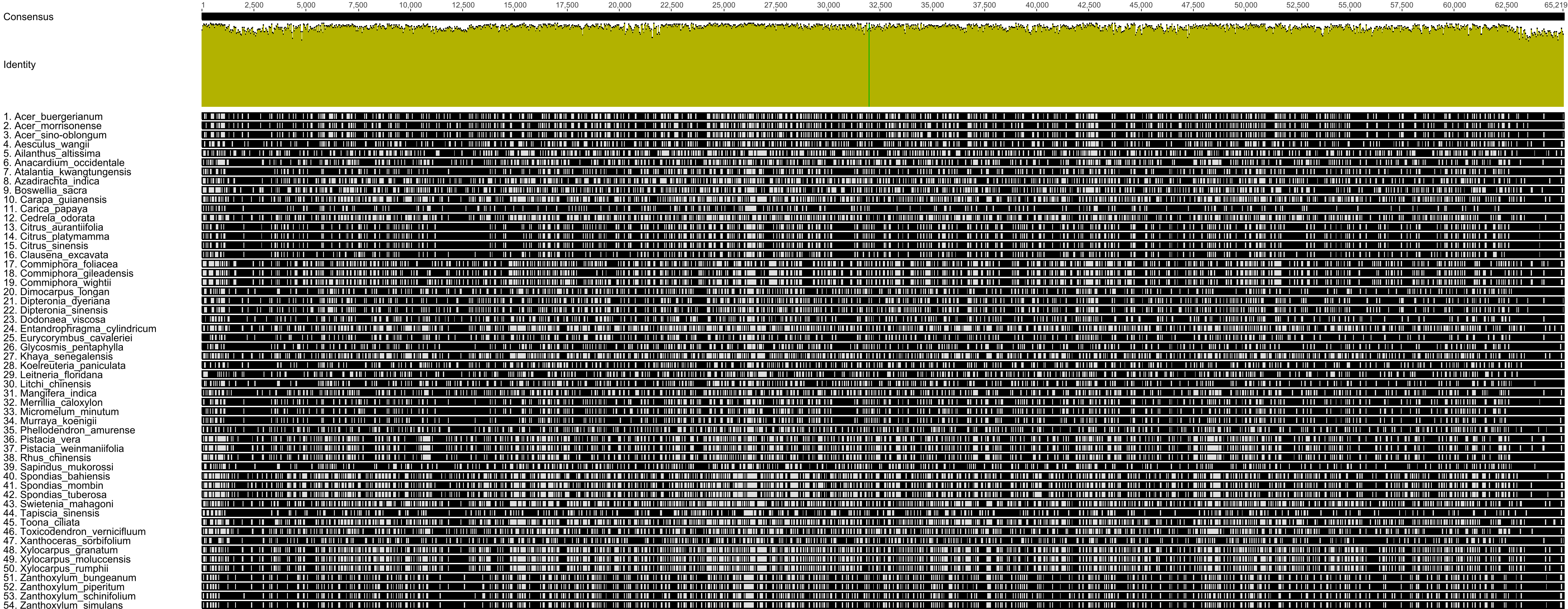

Supplement: Supplementary Materials — Figure S1: gene map and MAUVE alignment of five Anacardiaceae chloroplast genomes with Rhus chinensis removed. Figure S2: the linear correlation between the length of IR and the total length of the complete chloroplast genome sequence. Figure S3: the final alignment produced by the HomBlocks pipeline. Figure S4: visualization of genes that were integrated into the final alignment and their corresponding regions. Table S1: GenBank accession numbers of the complete chloroplast genome sequences of 52 species in Sapindales and two outgroups from Brassicales and Huerteales used for the phylogenetic analyses. Table S2: the best-fit partitioning schemes and DNA substitution models determined by PartitionFinder. Table S3: genes contained in the Toxicodendron vernicifluum chloroplast genome. Table S4: genes with introns in the Toxicodendron vernicifluum chloroplast genome. Table S5: the codon number and relative synonymous codon usage (RSCU) values calculated based on the coding sequences of 81 protein-coding genes in the complete chloroplast genome of Toxicodendron vernicifluum. Table S6: simple sequence repeats (SSRs) of the Toxicodendron vernicifluum chloroplast genome. Table S7: long repeats in the Toxicodendron vernicifluum chloroplast genome. Table S8: two single nucleotide variants between the complete chloroplast genome of Toxicodendron vernicifluum and T. vernicifluum cv. Dahongpao. [file 9014873.f1.zip › 9014873.f1/FigureS3.pdf]
